# A Decision Aid Intervention for Family Building After Cancer: Developmental Study on the Initial Steps to Consider When Designing a Web-Based Prototype

**DOI:** 10.2196/20841

**Published:** 2021-01-22

**Authors:** Catherine Benedict, Katherine L Dauber-Decker, D'Arcy King, Alexandria Hahn, Jennifer S Ford, Michael Diefenbach

**Affiliations:** 1 Stanford University School of Medicine Palo Alto, CA United States; 2 Stanford Cancer Institute Palo Alto, CA United States; 3 Donald and Barbara Zucker School of Medicine at Hofstra/Northwell Manhasset, NY United States; 4 Albert Einstein College of Medicine The Bronx, NY United States; 5 Hunter College and The Graduate Center City University of New York New York, NY United States

**Keywords:** patient-centered care, user-centered design, decision support techniques, decision aid, cancer, fertility, internet-based intervention, web-based intervention, mobile phone, psychosocial intervention

## Abstract

**Background:**

An important aspect of patient-centered care involves ensuring that patient-directed resources are usable, understandable, and responsive to patients’ needs. A user-centered design refers to an empathy-based framework and an iterative design approach for developing a product or solution that is based on an in-depth understanding of users’ needs, values, abilities, and limitations.

**Objective:**

This study presents the steps taken to develop a prototype for a patient resource for young women who have completed treatment for gonadotoxic cancer to support their decision making about follow-up fertility care and family building.

**Methods:**

User-centered design practices were used to develop *Roadmap to Parenthood*, a decision aid (DA) website for family building after cancer. A multidisciplinary steering group was assembled and input was provided. Guidelines from the International Patient DA Society and the Ottawa Decision Support Framework were used throughout the development process. In addition, guidelines for developing health DAs with respect to patient diversity and health literacy were also followed.

**Results:**

The *Roadmap to Parenthood* DA website prototype was systematically and iteratively developed. An extensive process of designing and developing solutions from the perspective of the end user was followed. The steps taken included formative work to identify user needs; determining goals, format, and delivery; design processes (eg, personas, storyboards, information architecture, user journey mapping, and wireframing); and content development. Additional design considerations addressed the unique needs of this patient population, including the emotional experiences related to this topic and decision-making context wherein decisions could be considered iteratively while involving a multistep process.

**Conclusions:**

The design strategies presented in this study describe important steps in the early phases of developing a user-centered resource, which will enhance the starting point for usability testing and further design modifications. Future research will pilot test the DA and a planning tool, and evaluate improvement in the decisional conflict regarding family building after cancer. Consistent with a patient-centered approach to health care, the strategies described here may be generalized and applied to the development of other patient resources and clinical contexts to optimize usability, empathy, and user engagement.

## Introduction

### Background

Patient-centered care is well established as an important aspect of health care quality. As put forth by the Institute of Medicine, all care should respect and be responsive to patients’ preferences, needs, and values [1]. Patients should have access to education and support to act as informed decision makers and participate in shared decision making with providers to ensure that their individual values are reflected in the treatment plans [2]. Operationally, an important aspect of patient-centered care involves ensuring that patient-directed information, education, and communication are usable, understandable, and responsive to patients’ needs. To support value-based decision making, it is important to develop patient resources with the target user group in mind.

This study focuses on oncofertility as an example of a clinical context in which there is an unmet need for patient-centered support. For young adult survivors of cancer (ie, aged 18-39 years), fertility is ranked among the most important survivorship issues [3,4]. Patient-centered resources are needed to inform patients about infertility risks and family-building options, support their decision making, guide their involvement in seeking care, and prepare them for potential future challenges. This paper describes the first phase of the development process of a patient decision aid (DA) and planning tool for family building after cancer.

### Family Building After Cancer

Owing to gonadotoxic treatments, many women experience reduced ovarian function or are unable to safely carry a pregnancy to term after cancer. The prevalence of primary ovarian insufficiency in female survivors of pediatric, adolescent, and young adult cancers ranges from 2% to 82%, based on patient factors, cancer diagnosis, and treatment exposures [5]. Alternative family-building options include the use of assisted reproductive technology, such as in vitro fertilization (IVF) and surrogacy, or adoption or fostering. With assisted reproduction, options comprise the use of fresh, frozen, or donated gametes to achieve pregnancy in the survivor or a gestational carrier. Adoption may be domestic or international. Each of these family-building options comes with a number of physical, emotional, financial, legal, and logistical challenges that need proper consideration; hence, decision making can be complex. For many patients, there may be benefits of an *early action* even if desired family building may be years away, including undergoing a fertility evaluation posttreatment to better understand their reproductive options and expected reproductive timeline, undergoing egg/embryo freezing posttreatment if they are at a risk for early menopause but not yet ready to start their family, or financial planning. Family-building decisions are based on values, and survivors must weigh the pros and cons of their options regarding risk-benefit tradeoffs. Given the emotional salience of motherhood desires, many women report high levels of uncertainty and distress when prompted to consider fertility and family-building decisions after cancer [6].

### Decision Support

Young female survivors of cancer report unmet support needs related to posttreatment fertility care in survivorship and want to be provided informational resources to help them understand their options for pursuing future parenthood [7,8]. Patient DAs are effective for improving tailored decision-making quality such that the users are more likely to be informed, gain clarity about how their values align with their decision options, and take a more active role in decision making [9]. Advantages of delivering patient DAs over the internet include an increasing reach and potential effectiveness [10]. Multiple patient DAs exist for young women diagnosed with cancer who are considering fertility preservation before treatment [11,12]. Although these studies support the use of DAs for fertility-related decisions in the context of cancer care [11,12], to our knowledge, there are no decision support resources that address the posttreatment reproductive survivorship care and family-building decisions that must be made after the completion of treatment.

### User-Centered Design

This study used user-centered design principles to develop a patient resource that supports decision making about family building after cancer treatment. A user-centered design is an empathy-based framework and an iterative design approach for developing a product or solution based on an in-depth understanding of users’ needs, values, abilities, and limitations. This iterative process is effective and essential because it places end users at the center of every stage of development—in this case young adult female survivors of cancer—to ensure that the end product reflects and addresses their needs [13]. Conversely, the failure to consider end users’ insights, feedback, and needs results in products and solutions that are less likely to achieve optimal adoption, retention, and advocacy [14]. Technology acceptance models and theories on telemedicine adoption highlight the importance of co-design with end users to develop products that are perceived as useful, easy to use, and responsive to needs [15,16].

### Study Objectives

To address a critical gap in young adult cancer survivorship care, we set out to develop a web-based patient DA and planning tool to support young women interested in family building after cancer. This study presents the steps taken to develop the prototype of the website, *Roadmap to Parenthood*, based on user-centered design practices and guidelines for developing DAs and health care resources for diverse patient groups and health literacy levels. This work was guided by a theoretical approach grounded in the self-regulation theory [17,18] and further developed in our preliminary work, which is described elsewhere [19,20]. In this paper, we review the *initial* design steps and process to develop a DA prototype before conducting formal usability testing. These steps aim to optimize usability, empathy, and user engagement to ensure universal applicability across patient subgroups. Our intention in this paper is to thoroughly describe the prototype design process, which allows us to enter a formal usability testing phase that considers key design issues and user feedback.

## Methods

### Preliminary Studies

The study team led several oncofertility studies focusing on young adult female cancer survivors’ experiences related to fertility and family building posttreatment and identified unmet decision-making needs and patient preferences for support. Our national survey of posttreatment reproductive concerns and decision making uncertainty identified the areas of decisional conflict about family building after cancer (eg, lack of information, clarification of values, and lack of emotional support) [6]. Two additional qualitative studies explored posttreatment fertility concerns [21] and family-building experiences [22] and informed our understanding of user needs. On the basis of this work, semistructured interviews (N=25) were conducted with young adult female survivors of cancer (aged 15-45 years) who received gonadotoxic treatment and were either interested in future family building or uncertain about their family-building plans [19]. Briefly, women reported high rates of unmet information needs, including uncertainty about reproductive survivorship care and where to obtain trusted information. They felt overwhelmed and distressed by the prospect of pursuing family building and its expected, associated challenges [19]. When asked about support preferences, they indicated a desire for *step-by-step* instructions to learn about their options and guide decision making and follow-up care [20]. They also reported a preference for web-based resources for self-education, which they envisioned would prepare them for and provide complementary support to in-person counseling with a clinician [20]. Notably, although the definition of *young adult* per the National Cancer Institute (NCI) is defined as an individual aged between 18 and 39 years, our work included women aged 15 to 45 years, as fertility and family-building concerns are highly relevant at somewhat younger and older ages [23,24].

### Study Design

**Figure 1:**
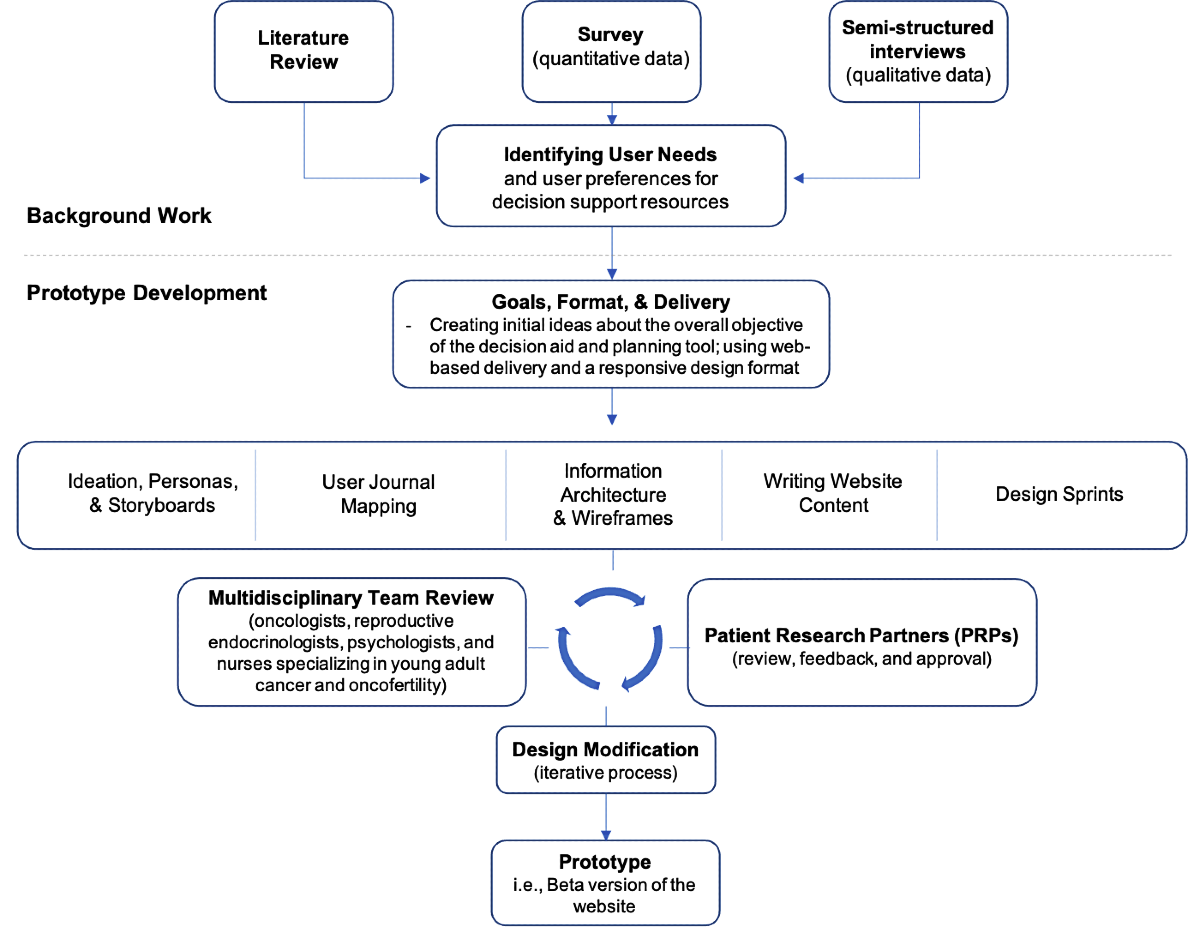
Steps taken to develop a patient decision aid and planning tool prototype using user-centered design strategies. Adapted from the user-centered design process map from the National Institutes of Health.

The *Roadmap to Parenthood* DA and planning tool (website) was developed by following the steps depicted in Figure 1. The website was designed and tailored considering the shared experiences, emotions, and support needs of young adult female survivors of cancer identified in our previous work. All procedures followed user-centered design methods such that users’ needs, contexts, and points of view were key drivers in the iterative design decisions throughout product development [15]. For this stage of the development process, decisions were made with input from patient research partners representing the target user population with the goal of optimizing the prototype design to best prepare for usability testing (which is currently underway). The development process followed the guidelines set forth by Coulter et al [25] and was consistent with the International Patient Decision Aid Society (IPDAS) and the Ottawa Decision Support Framework guidelines for patient DAs [25-28]. For the purposes of building a website, guidelines from the Department of Health and Human Services were followed for best practices of a user-centered web design and digital communication [29].

### Exploratory Work

The research team reviewed and discussed oncofertility patient DAs, web-based oncofertility open access resources, and websites targeting young women such as those focused on women’s health and fertility to explore ideas about structure, tonality, and appealing visual identity and design aesthetic for this demographic (Multimedia Appendix 1). The research team then completed a *discovery worksheet* to ensure alignment with the web developer regarding the goals of the project (Multimedia Appendix 2).

### Responsive Design Format

We selected a digital format to optimize user access, flexibility, and convenience, aligned with the stated preference of the target user group [20]. Internet use is nearly ubiquitous in the United States among young adults (eg, 97%-100%) with 77% of adults aged 18 to 29 years having home broadband service and 96% owning a smartphone [30-32]. A responsive design website was developed for the decision tool. This choice was made given the flexibility of adapting the layout and content across digital devices and the relative ease and low cost of website updates. A responsive design also provides a consistent user experience regardless of the operating system or device—desktop computer/laptop, tablet, or mobile phone.

### Steering Group

A multidisciplinary steering group was assembled, which included clinicians and researchers with expertise in oncofertility and developing patient DAs (ie, oncologists, reproductive endocrinologists, psychologists, and nurses), a digital health communication researcher, an expert in user-centered design and usability testing, and a web developer. The team also included 4 patient research partners, who were asked for advice, provided feedback, and reviewed design decisions and content throughout the ideation phases and the entire development process.

## Results

The following steps were taken to develop the prototype website of *Roadmap to Parenthood*. The tool was designed to be used by young adult female survivors of cancer who completed gonadotoxic treatment and were interested in future family building or were uncertain of their family-building plans. The primary purpose of the tool was to educate users about options to achieve parenthood after cancer (ie, natural conception, IVF or surrogacy with fresh/frozen/donated gametes, and adoption or fostering) and to guide value-based decision making and preparatory action toward family-building goals.

### Identifying User Needs

The first phase of user-centered design processes involves exploratory work to fully understand and define the problem, comprising literature reviews, end-user interviews and surveys, and team brainstorming [13]. We did much of this work previously, and user needs are described under *preliminary studies.* We also conducted a scoping review of the literature [11,12] and discussed our understanding of user needs with our patient research partners. Common themes (eg, lack of information, uncertainty about reproductive survivorship care options, and a lack of awareness about high costs and legal complications) were reviewed by the research team, which led to brainstorming about how a web-based decision support and planning tool could address user needs (described in the following sections).

### Determination of Goals, Format, and Delivery

From the start, the overall objective of the site was to help users become informed, clarify values and priorities with respect to family-building goals, and consider options for actionable preparatory behaviors (eg, pursuing a fertility evaluation or accessing social support). Family-building decision options included the possibility of natural conception and alternative options, that is, IVF, surrogacy, and adoption with subsidiary options (ie, use of fresh, frozen, or donor gametes and domestic or international adoption or fostering). Notably, personalized information about infertility risk and likelihood of success with family-building options could not be provided. Instead, the tool was built to increase awareness of the potential for challenges and benefits of early action and to prompt decisions about pursuing *next steps* aligned with parenthood goals. At the same time, we aimed to create a website that would *feel* empowering and would be usable, engaging, and effective. The tool was designed to be used independently by young adult female survivors of cancer and delivered via internet access using a responsive design format.

### Design Process

We designed the DA website using an agile development process, which provided a nimble system for ongoing revision and iterative design decision making based on team review and input from the web developer, usability experts, and patient research partners. Modified beginning stage *design sprints* (ie, a rapid cycle user-centered prototype development and testing process [33]) were undertaken to generate and test ideas, obtain feedback, and iterate features of the prototype. Patient research partners were asked via email and phone/video communication for feedback and recommendations.

#### Ideation Phase

Ultimately, we wanted the website to be empowering for young women by supporting their family-building decision-making processes. A period of research and brainstorming was undertaken by the team with input from the web developer and patient research partners. We reviewed 9 publications reporting on 7 oncofertility patient DAs and were able to access 5 of the DAs available on the web (Multimedia Appendix 1). We looked for IPDAS DA components and descriptions of development processes. We discussed aspects we believed were useful, appealing, and should be considered for our own design and, conversely, aspects that we felt could be improved upon. For example, very dense text and long paragraphs prompted discussions about layout and content organization. It was also our aim to create a website that would feel approachable while conveying trustworthiness and reliability (ie, the *personality* of the website). A review of web-based oncofertility resources and women-targeted websites (Multimedia Appendix 1) focused on the esthetic appeal of designs and tonality. The websites rated most positively were those that felt *clean* and easy to use, with clear text and appealing use of graphics and white space. Fewer favorably reviewed websites included elements that felt stereotypically feminine (eg, *too pink*), content that felt crowded, or pages that had distracting visual designs such as overlaying text on busy backgrounds. Patient research partners were asked for feedback about the likes and dislikes and ideas for an appealing *look and feel* of DA and website esthetics. Tonality across DAs and websites ranged from professional and more business-like to friendly and more conversational. We aimed to strike a balance between friendly and approachable, yet informative and trustworthy.

We completed the *discovery worksheet* to facilitate communication and a shared understanding among team members about the overall objective for the site and initial stylistic ideas (Multimedia Appendix 2). For example, the 4 stylistic descriptors of the ideal website were empowering, informative, friendly, and clean. The web developer used the worksheet and descriptions of our likes/dislikes of the DA/website examples to understand the overall objective for the website and design, combined with user personas that provided further guidance.

#### Personas and Storyboards

**Figure 2:**
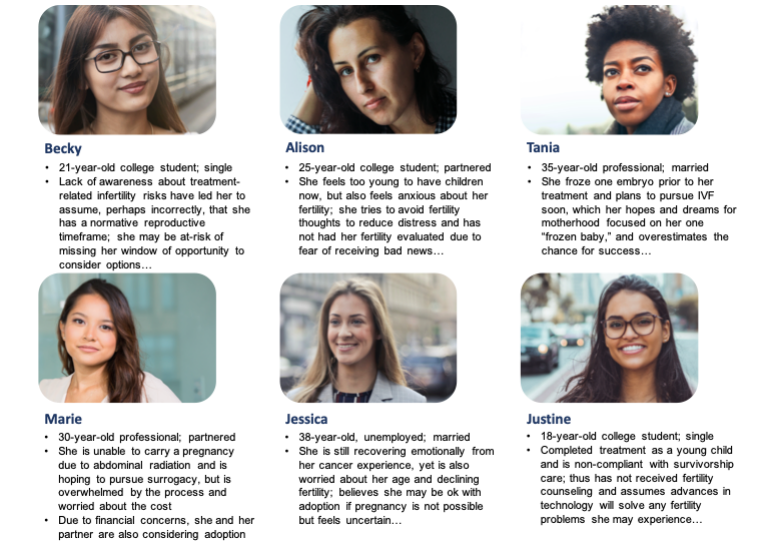
Personas depicting “user types” for the decision aid and planning tool website. Shortened versions of personas are depicted. IVF: in vitro fertilization.

On the basis of our background work [19-22], literature review [34], and clinical experience (Figure 2), 6 personas were created representing *user types*. The personas varied to represent different patient situations based on sociodemographic characteristics, cancer, and reproductive health factors. These factors were used to construct an overall picture of user archetypes and the kinds of questions, concerns, intentions, and goals they would have to interact with the website. Each persona was given a name, sociodemographic descriptors, a cancer story, social context, and description of values, priorities, and goals related to family building. Personas depicted the types of users for which the website was being designed and the scope of user intentions and needs. Decisions about design, features, and navigation were made to meet the needs of all user personas, in combination with the discovery worksheet.

Storyboarding was used in conjunction with persona mapping to envision the end-to-end user experience of someone engaging with the site over time. This process mapped how the website would fit into a user’s life as well as in what context they would be seeking out and engaging with the site for the first time and in subsequent viewings. We based our storyboards on the lives of actual patients depicted through the personas, imagining their life experiences leading up to and after viewing the website. For example, a young woman in her early 20s, not yet ready to have children but aware that she may be at risk for fertility problems in the future, may approach the website with curiosity to learn about her options and recommendations for reproductive health as a survivor of cancer. Alternatively, an older woman in her late 30s, who is ready to have a child and fearful that known fertility problems will prevent her from becoming a mother, may approach the site with greater anxiety and fear, concern about reproductive time pressure, and looking for guidance for immediate action and resources.

#### User Journey Mapping

User journey mapping involved envisioning the different ways in which users might navigate through our website. During the user journey mapping, it is important to consider what the user will be thinking, feeling, and doing as they engage with the website. The personas we created guided our vision for user journey options. For example, given our conceptualization of users having different levels of knowledge and decision-making readiness at the outset, we debated various options for progressing through the website. The goal of user journey mapping was to plan and optimize how users would move through the website, identify gaps in the user experience, and iteratively pivot to correct errors [35].

#### User Content Control

It was important to design the website giving users control over their user journey with freedom to access web pages that best matched their needs, as opposed to a more rigid user journey with a single preconceived path through content (ie, similar to paper-based resources in which there is only one path to access content by turning pages). Content control is intended to provide users with control over the order, detail, and type of evidence presented. Providing users greater content control is related to improved quality of decision making [36]. Conversely, tailoring content via preset frameworks has been shown to reduce decision-making quality, despite the intention that more personalized information will be delivered to the user [36,37]. On the basis of this research and as depicted in the personas and storyboarding, we sought to give users a greater control over their user journey to explore content and review material relevant to their situations and interests.

We imagined that some users would need to move linearly through the DA components, starting with education about fertility and cancer treatment effects and moving on to review information about family-building options, whereas others may be quite informed and ready for *next steps* (eg, questions to ask your doctor) and still others may wish to avoid information that is irrelevant or even upsetting if particular family-building options are no longer possible. We wanted to allow flexibility to navigate through the website to best meet different users’ needs and motivations. One idea was to prompt users to answer a set of questions on the homepage with a branching logic to guide them to the best landing page, but this was discarded after initial mockups because of its complexity. Ultimately, we created an omnichannel user journey, or *choose your own adventure* design, in which users had control over the user journey and could easily see options for *next step* recommendations and click on webpages to access content that matched their needs, preferences, and decision-making readiness. The DA components were marked across the top navigation bar. Many pages included links at the bottom that suggested the next pages to visit but these could be ignored, and the user had control over which pages they visited. This was an iterative process designed to match users at different stages of decision making. We identified gaps in our initial design ideas and developed solutions to optimize the user experience.

#### Information Architecture

**Figure 3:**
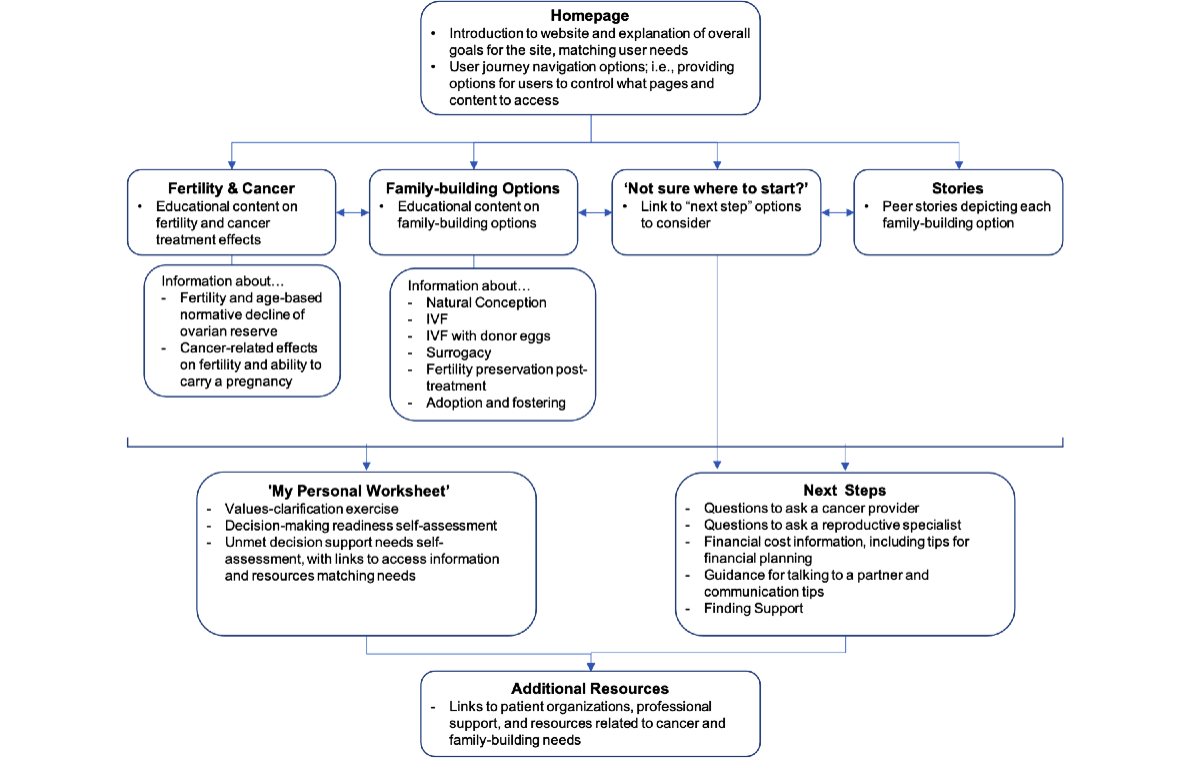
Depiction of the information architecture of the decision aid website. IPDAS: International Patient Decision Aid Society.

Following the development of user journeys, we moved into the information architecture phase of the project (Figure 3). This process involved leveraging the user flows to decide how content should be organized, structured, and labeled across the website pages. The main components of the information architecture process included finalizing decisions about categorizing and structuring information, labeling systems (ie, how information is represented), navigation systems (ie, how users would browse or move through information), and search systems (ie, how users would look for and find information). Various ways of organizing content and implications for the user journey have been discussed and debated. For example, multiple options were considered for how to best introduce and categorize family-building options and how to organize decision support content. As depicted in Figure 3, users had multiple options to move on from the homepage. The *next step* options were grouped together and introduced to users on a single page with links through which they could click for more content on each topic. Decisions about the information architecture guided content strategy and informed the design of the user interface, which was later used in wireframing and prototyping.

#### Sketches and Wireframes

The next step in our website development was to create sketches of our ideas and then wireframes. All appropriate web standards were used to develop content for the site. Our overarching goal with the content was to make it user-friendly and helpful to the reader. We placed special emphasis on using concrete examples that would be highly relevant to the reader. Initial sketches were made by the study team via pen and paper and dry-erase white boards, and ideas were discussed with the web developer. Wireframes (ie, two-dimensional models of the website interface) focused on content presentation and space allocation, functionalities of the site, and intended behaviors of the user [38]. They were used to give the team a sense of how the site would be organized and function once it was fully developed, without focusing on styling, color, and graphics. These digital wireframes allowed us to collect early feedback from collaborators and patient research partners and led to multiple iterations of website wireframes. Patient research partners were critical at this juncture to clearly understand the purpose of the website, comment on available content, design esthetic upfront, and appropriate navigation that would support their decision-making needs. Once the basic wireframes were set, more detailed illustrations were created using static images to depict the *look and feel* of the site, such as color palettes, font choices, and pictures. They discussed the emotional experiences that users might have when approaching the website, based on the stress and uncertainty of fertility and family-building futures, and cited a desire for the website to feel *calming* and *hopeful*. Wireframes were informed by this feedback and the work done during the *discovery phase*.

#### Aligning on a Design Aesthetic

It is recommended to use images that end users will find to be realistic and relatable when designing health- and medicine-related content [29]. Research suggests users of digital health tools may prefer photographs of *real* people, as opposed to illustrations or no photographs at all [39]. It is also important to show people of diverse backgrounds, allowing more people to *find themselves* and relate to the content [40]. Accordingly, we included photographic images of young women representing different races, ethnicities, and ages throughout the site. There were no medical or fertility-related photographs. To facilitate understanding, whenever possible, we accompanied written text with graphs (eg, a line graph depicting the decline in ovarian reserve over time) and comparison charts (eg, a table with the costs of family-building options listed side by side). Each family-building option had a different icon to guide the user’s journey and comprehension. The research team developed an initial conceptualization of photos, icons, and graphs based on the dual goals of optimizing usability and achieving the desired stylistic feel, and patient research partners were asked for their impressions. Generally, feedback was positive; however, designs were modified based on specific suggestions (eg, to use a different photo or improve labeling). One key issue was how to best depict potentially distressing information. In particular, patient research partners told us that seeing the downward slope of the line graph showing a declining ovarian reserve over time was a powerful and potentially upsetting image. The pros and cons of conveying this information in text or graphs were discussed. Ultimately, with agreement from our patient research partners, we decided to keep the graph for its effectiveness in displaying the critical information. We will test these design decisions and their emotional impact during usability testing.

### Content Development

In developing the narrative for the website, guidelines for developing web-based informational content were followed [41]. Writing user-friendly content for a website involves consideration of word choice (eg, use of *plain* *language* and keywords known to users and an active voice), use of short sentences and paragraphs, chunking content and presenting information in bullets and numbered lists, use of clearly distinguished headlines and subheadings, placement of key informational pieces on the page, and use of white space [41,42]. We followed the plain language checklist for writing website content (Textbox 1; adapted from the checklist developed by the National Institutes of Health [NIH; 43]). Definitions of medical terminology were provided, and simpler medical terms were used whenever possible. For example, the title *ask a fertility specialist* replaced *ask a reproductive endocrinologist*, and the definition for a reproductive endocrinologist was provided for reference.

**Plain language checklist for writing for the web.**

1. Be concise; eliminate unnecessary words
2. Break information up into separate topics
3. Use short paragraphs (ie, shorter paragraphs than when writing for printed materials)
4. Use short lists and bullets to organize information
5. Use headings and subheadings that are descriptive, with limited text under each heading
6. Consider using questions as headings
7. Present each topic or point separately
8. Keep the information on each page to no more than two levels
9. Use white space to allow users to easily scan the page for key information
10. Write using the same words users would use when doing a web search for the information, particularly for page titles and headings
11. Clearly explain things such that each page can stand on its own; ie, don’t assume users will have knowledge of the subject or have read other content/pages on the site
12. Use language to guide the user journey that describes what the user will get if they click on the link; ie, never use “click here” as a link
13. Eliminate unnecessary words as much as possible

Content was also written to be all-inclusive with respect to user diversity, particularly regarding partnership status (ie, single vs coupled users), sexual orientation (ie, users identifying as lesbian, gay, bisexual, transgender, and queer/questioning [LGBTQ]), and definitions of family makeup (eg, same-sex parents and single women pursuing parenthood). Users were not assumed to have a partner (now or when pursuing family building), and partners were not assumed to be of a specific gender. Listed resources provided access to more detailed information (eg, state-by-state laws regulating LGBTQ and same-sex couple adoption, legal advocacy, and financial grant opportunities for LGBTQ prospective parents). Religious and cultural factors that may impact users’ decision making, particularly with respect to the use of reproductive medicine (eg, transvaginal procedures and creation of embryos in the lab), were addressed in a limited way at several points throughout the website. For example, users are prompted to consider religious, cultural, and ethical beliefs in relation to their decision-making options in the values clarification exercise and can also reflect on these factors when answering open-ended questions.

### Additional Design Considerations

Patient research partners discussed the emotional experiences that users might have when approaching the website, based on the stress and uncertainty of fertility and family-building futures, and cited a desire for the website to feel *calming* and *hopeful*. Several design considerations were made to reflect the emotional experiences and health literacy levels of users interacting with the site.

#### Designing for Iterative Decision Making

Most patient DAs for health care decisions involve discrete periods in which a single decision about treatment options must be made [43]. DAs developed in cancer and fertility have almost exclusively focused on pretreatment fertility preservation in which there are 2 decision options (yes or no) and a limited time window, as cancer treatment must be initiated [12,44]. Conversely, decision making about family building may involve a more complex set of decision points. For example, for some users, the focus of the decision may be about seeing a fertility specialist, and decision options may change based on feedback about reproductive viability and the likelihood of success with natural pregnancy, IVF, or surrogacy, thus changing their informational and support needs. Many survivors may first prioritize having a biologically related child, but if given a low chance for success, they may re-evaluate their priorities and choose to spend financial resources on pursue adoption. Others may restart the decision process if they are unsuccessful, such as after failed IVF attempts, or if the challenges become too great. Single women may change their preferences when they involve a decision partner. On the basis of this conceptualization of longitudinal decision-making processes, the design of the website included support for iterative engagement such that the information architecture was set up to allow users to have maximum control over the user journey and easily circle back to content about alternative family-building options.

#### Emotional Design

Our previous work suggested that women who experienced more intense emotions of distress and fear often described lower self-efficacy to manage risks and, at the most extreme, avoidance of fertility information and disengagement from decision-making processes [19]. These findings are consistent with the research delineating the impact of affective states on medical decision making and behavior, such that anxiety and fear tend to lead individuals to prioritize short-term gratification over long-term goals [45,46]. In this case, young women who are distressed about infertility risks or fear of receiving bad news may avoid information to avoid further distress (thus prioritizing short-term relief), diminishing their chances of achieving long-term goals for parenthood. One of the objectives for the website was to be empowering for young women, for example, to guide users in becoming informed and setting realistic expectations about potential challenges, while inspiring hope and optimism that parenthood may be achieved. With consultation and input from experts on the team, we aimed to achieve this emotional experience for users through design decisions about tonality, color, language, and picture selection. Acknowledging that information on the website may be upsetting for users, we made decisions about design and photo images to create a positive user experience (eg, facial expressions of women in photos that suggest confidence, hope, and optimism), without negating the difficulties and negative emotions users may experience as a part of this journey. We also used color and design to facilitate comprehension and guide engagement with the site. We attempted to avoid design elements that might overwhelm users, perhaps leading them to abandon the website. For example, large blocks of text can be difficult to read and comprehend, which may be even more challenging for cancer survivors with lasting treatment side effects such as fatigue or cognitive impairment [47], and our patient research partners corroborated concerns about information overload and text-heavy pages. Efforts to reduce the cognitive load and emotional distress included using short text blocks, white space, clearly identified and defined terminology, and graphs, charts, and icons.

### Guidelines and Standards

The website was designed to meet varying health literacy and reading levels of users and in accordance with the IPDAS guidelines and the Ottawa Decision Support Framework for developing patient DAs [25,26,48], and health literacy guidelines, including those set by the NIH [49,50]. The Centers for Medicaid and Medicare Services *toolkit for making written material clear and effective* was also employed [51]. The IPDAS checklist is presented in Table 1, whereas the Health Literacy Online Strategies Checklist is presented in Table 2. Standards required for the design and development of websites affiliated with the US Department of Health and Human Services were also reviewed and used to guide design decisions [52]. Guidelines from the Office of Disease Prevention and Health Promotion [49], NIH [50], and the Centers for Medicare and Medicaid Services [51] for designing digital health websites and information tools for low health literacy and culturally diverse populations were also followed.

**Table 1:** Review of the Roadmap to Parenthood patient decision aid using the International Patient Decision Aid Standards quality checklist.

Criteria		Answer
**Criteria to be defined as a patient DA^a^**		
	1. The DA describes the condition related to the decision	Yes
	2. The DA describes the decision that needs to be considered	Yes
	3. The DA identifies the target audience	Yes
	4. The DA lists the options (health care or other)	Yes
	5. The DA has information about the positive features of the options (eg, benefits or advantages)	Yes
	6. The DA has information about the negative features of the options (eg, harms, side effects, or disadvantages)	Yes
	7. The DA helps patients clarify their values for outcomes of options by (a) asking people to think about which positive and negative features of the options matter most to them AND/OR (b) describing each option to help patients imagine the physical, social, and/or psychological effects^b^	Yes
	8. The DA makes it possible to compare the positive and negative features of the available options	Yes
	9. The DA shows the negative and positive features of the options with equal detail	Yes
	10. The DA compares probabilities (eg, chance of a disease, benefit, harm, or side effect) of options using the same denominator	N/A^c,d^
	11. The DA (or available technical documents) reports funding sources for development	Yes
	12. The DA reports whether authors of the DA or their affiliations stand to gain or lose by choices people make after using the DA	Yes
	13. The DA includes authors/developers’ credentials or qualifications	Yes
	14. The DA reports the date when it was last updated	Yes
	15. The DA (or available technical document) reports readability levels	Yes
	16. The DA provides references to scientific evidence used	Yes
**Other criteria for Das about screening or testing**		
	17. The DA has information about what the test is designed to measure	Yes^e^
	18. The DA describes possible next steps based on the test results	Yes^e^
	19. The DA has information about the chances of disease being found with and without screening	Yes^e^
	20. The DA has information about detection and treatment of disease that would never have caused problems if screening had not been done	Yes^e^
**Other criteria indicating quality**		
	21. The DA describes what happens in the natural course of the condition (health or other) if no action is taken	Yes
	22. The DA has information about the procedures involved (eg, what is done before, during, and after the health care option)	Yes
	23 The information about outcomes of options (positive and negative) includes the changes that may happen	N/A^d^
	24. The DA presents probabilities using event rates in a defined group of people for a specified time	N/A^d^
	25. The DA compares probabilities of options over the same period of time	N/A^d^
	26. The DA uses the same scales in diagrams comparing options	Yes
	27. Users (people who previously faced the decision) were asked what they need to prepare them to discuss a specific decision	Yes
	28. The DA was reviewed by people who previously faced the decision and were not involved in the DA’s development and field testing	Yes
	29. People who were facing the decision field tested the DA	No^f^
	30. Field testing showed that the DA was acceptable to users (the general public and practitioners)	No^f^
	31. Field testing showed that people who were undecided felt that the information was presented in a balanced way	No^f^
	32. There is evidence that the DA (or one based on the same template) helps people know about the available options and their features	N/A^g^
	33. There is evidence that the DA (or one based on the same template) improves the match between the features that matter most to the informed person and the option that is chosen	N/A^g^

^a^DA: decision aid.

^b^We expanded this definition to also include financial effects of decision option outcomes.

^c^N/A: not applicable.

^d^The primary purpose of the decision aid is to educate and support patients facing limited family-building options in which it is not possible to predict the likelihood of success or failure with in vitro fertilization, surrogacy, and adoption.

^e^The decision aid and planning tool provides information on infertility risk due to gonadotoxic cancer treatment, options to test fertility, and possible next steps for family building based on the results of fertility testing and indications of reproductive potential; however, this is only one aspect of the entire decision-making process encompassing family building after cancer.

^f^This criterion was not yet relevant, as the patient decision aid was still in development. Usability and field testing are currently underway.

^g^Study of efficacy will begin after the completion of usability and field testing and once the design of the decision aid is finalized.

**Table 2:** Review of the Roadmap to Parenthood patient decision aid using the Health Literacy Online Strategies Checklist from the National Institutes of Health.

Criteria		Answer
**Write actionable content**		
	1. Identify user motivations and goals	Yes
	2. Put the most important information first	Yes
	3. Describe the health behavior [information] – just the basics	Yes
	4. Stay positive. Include the benefits of taking action	Yes
	5. Provide specific action steps	Yes
	6. Write in plain language	Yes
	7. Check content for accuracy	Yes
**Display content clearly on the page**		
	8. Limit paragraph size. Use bullets and short lists	Yes
	9. Use meaningful headings	Yes
	10. Use readable font	Yes
	11. Use white space and avoid clutter	Yes
	12. Keep the most important content above the fold – even on mobile	Yes
	13. Use links effectively	Yes
	14. Use color or underline to identify links	Yes
	15. Use Images that help people learn	Yes
	16. Use appropriate contrast	Yes
	17. Make web content printer-friendly	Yes
	18. Make your site accessible to people with disabilities	Yes^a^
	19. Make websites responsive	Yes
	20. Design mobile content to meet mobile users’ needs	Yes
**Organize content and simplify navigation**		
	21. Create a simple and engaging homepage	Yes
	22. Label and organize content with your users in mind	Yes
	23. Create linear information paths	Yes^b^
	24. Give buttons meaningful labels	Yes
	25. Make clickable elements recognizable	Yes
	26. Make sure the browser “back” button works	Yes
	27. Provide easy access to home and menu pages	Yes
	28. Give users options to browse	Yes
	29. Include a simple search function	No^c^
	30. Display search results clearly	No^c^
**Engage users**		
	31. Share information through multimedia	No
	32. Design intuitive interactive graphics and tools	Yes
	33. Provide tailored information	Yes^d^
	34. Create user-friendly forms and quizzes	Yes
	35. Consider social media sharing options	N/A^e,f^
**Test your site with users with limited literacy skills**		
	36. Recruit users with limited literacy skills and limited health literacy skills	No^f^
	37. Identify and eliminate logistical barriers to participation	Yes
	38. Create plain language testing materials	Yes
	39. Test whether your content is understandable and actionable	No^f^
	40. Use moderators who have experience with users with limited literacy skills	N/A^g^

^a^Design decisions were made within the scope of the project to make content accessible to people with disabilities, including using large font and white space.

^b^Beginning sections of the decision aid tool were designed so that users would follow a linear path to obtain information about fertility, infertility risks associated with cancer, and family-building options. Following this, users were prompted to choose their own path with respect to which content was most applicable to their situation and needs (eg, finding support vs financial planning strategies).

^c^We were unable to include a search function due to the limitations of web development resources.

^d^Information was tailored to the extent that users had control over content they viewed because of the choose your own path click-through user journey design of the website, use of a drawer design to hide/reveal content based on user interest, and available drop-down features.

^e^N/A: not applicable.

^f^This criterion was not yet relevant, as the patient decision aid was still in development.

^g^The tool is designed to be used by young adult female cancer survivors independently, without help from moderators, clinicians, or professionals to provide guidance or decision support. Future work will explore options for use of the tool during patient-provider interactions and for shared decision making.

## Discussion

User-centered design practices involve an extensive and iterative process of designing and developing solutions from the perspective of the end user. The development of the *Roadmap to Parenthood* prototype was based on pilot work to understand the experiences and needs of young adult female survivors of cancer related to family building after cancer, combined with a collaborative, multidisciplinary team approach to making initial design decisions that would best meet their needs. Ultimately, usability testing with members of the target patient population is necessary to determine whether design decisions have been made to optimize the ease of use, comprehension, and usefulness of the product or whether improvements are needed. Once completed, we hope that this DA for family building after cancer will be a complementary resource to the DAs and resources that exist for pretreatment fertility preservation [11,12]. We followed similar development procedures to those reported for other DAs in the literature, including adherence to IPDAS guidelines, review of previously published DAs, use of a multidisciplinary team approach, iterative design with feedback from target users, and digital platforms [53-57]. Fertility preservation DAs have been shown to be acceptable and beneficial to young adult female cancer survivors [53,55,57], suggesting the DA presented here may similarly support patients through the next steps of family building.

The early design considerations presented here are important steps for developing a user-centered prototype that is a good starting point for usability testing. We are currently underway in conducting usability testing with target end users to obtain feedback about the website prototype. This process involves quantitative and qualitative data analytic approaches using standardized usability testing procedures including think-aloud sessions and validated usability assessment measures. Design modifications and additional testing will be conducted until user feedback indicates that we have optimized the product design with the appropriate degree of compassion and empathy. Following usability testing, we will conduct a single-arm pilot study to test the tool as a DA intervention for family building after cancer [58]. For this study, we will follow the SUNDAE Checklist (Standards for UNiversal reporting of patient Decision Aid Evaluations) for reporting patient DA evaluative studies [48]. Future directions of this research will also explore how the tool may be used for dyadic decision making, including users’ partners, and as a part of cancer survivorship care to support patient-provider communication and shared decision making.

### Limitations and Future Directions

There were limitations to this study. One of our most difficult tasks in creating the website was to balance the amount of informational content with concerns about *information overload*, an issue that was brought up by our patient research partners. Although the website provides a comprehensive overview of fertility and family-building topics and multiple aspects of decision-making support, some subsidiary topics were not as thoroughly reviewed. For example, while users are prompted to self-reflect and explore personal factors that are most relevant to their decisions, in-depth content specific to cultural and religious factors was limited. Similarly, this version of the website does not mention step-motherhood as a family-building option. Future usability testing will determine whether more comprehensive information is needed on these topics. In addition, while we decided to build a web-based resource to increase access and convenience among the target user group of young adult women who reported a preference for digital resources, we recognize that some members of the target population may not have regular or dependable access to the internet. However, we do not believe this is a widespread issue, based on the data gathered on internet accessibility across various demographic cohorts [30-32]. In order to accommodate users who may prefer a paper-based version, we included a print button on the top of each page that autoformats the website content for the ease of printing.

### Conclusions

Following this first phase of the development process of a patient DA and planning tool for family building after cancer, our subsequent usability testing phase will guide modifications and finalization of the design. This clinical research will contribute to a priority area set forth by the NCI and the American Society of Clinical Oncology to develop age-specific resources for young adult cancer survivors while leveraging the advantages of digital communication technology [59,60].

## Appendix

Multimedia Appendix 1 List of oncofertility patient decision aids, online resources, and websites reviewed during the ideation phase of website development.

Multimedia Appendix 2 Discovery worksheet for initial website design.

## Abbreviations

ASCO: American Society of Clinical Oncology
DA: decision aid
IPDAS: International Patient Decision Aid Society
IVF: in vitro fertilization
LGBTQ: lesbian, gay, bisexual, transgender, and queer/questioning
NCI: National Cancer Institute
NIH: National Institutes of Health
