# Supplementary material for: A Decision Aid Intervention for Family Building After Cancer: Developmental Study on the Initial Steps to Consider When Designing a Web-Based Prototype
Source: JMIR Form Res. 2021 Jan 22;5(1):e20841. doi: 10.2196/20841 (PMC7864768; doi:10.2196/20841)
Supplement: Multimedia Appendix 1 [file formative_v5i1e20841_app1.docx]

**Appendix 1.** List of oncofertility patient decision aids, online resources, and websites reviewed during the Ideation Phase of website development.^a^

| **Oncofertility Decision Aids** |  | |  | | |  | | | |
| --- | --- | --- | --- | --- | --- | --- | --- | --- | --- |
| **Patient Decision Aid (PtDA)^b^** | **Author** | | **Type** | | | **Country** | | | |
| ‘FertiEll’P’ | Benoit et al.[1] | | Web-based | | | France | | | |
| PtDA for Fertility Preservation | Ehrbar et al.[2,3] | | Website | | | Switzerland | | | |
| PtDA for Fertility Preservation | Peate et al.[4,5] | | Booklet | | | Australia | | | |
| PtDA for Fertility Preservation | Garvelink et al.[6] | | Website | | | Netherlands | | | |
| ‘Cancer, Fertility, & Me’ | Jones et al.[7] | | Website | | | United Kingdom | | | |
| ‘BEFORE’ (Begin Exploring Fertility Options, Risks, and Expectations) | Speller et al.[8] | | Website | | | Canada | | | |
| Pathways | Woodard et al.[9] | | Website | | | United States | | | |
| **Oncofertility Open Access Resources** | |  |  | | | | | |  |
| **Name** | **Development Group** | | | | | | **Type** | | |
| Female Fertility and Cancer | American Cancer Society | | | | | | Webpage | | |
| Fact Sheet | American Society of Reproductive Medicine | | | | | | Webpage | | |
| PtDA for Fertility Preservation | Breast Cancer Now | | | | | | Downloadable booklet | | |
| ‘Having a Baby After Cancer’ | Cancer.Net | | | | | | Webpage | | |
| Educational resources | Fertile Future | | | | | | Website | | |
| ‘Becoming a parent after cancer’ | LIVESTRONG | | | | | | Website | | |
| Educational resources, blog stories, and expert opinions | Living Beyond Breast Cancer | | | | | | Website; Webinars | | |
| ‘Building Your Family After Cancer Treatment: Information for Women’ | Memorial Sloan Kettering Cancer Center | | | | | | Webpage | | |
| Cancer and Fertility | National Comprehensive Cancer Network | | | | | | Website | | |
| ‘Learning about Cancer and Fertility’ | Oncofertility Consortium | | | | | | Website, Booklets | | |
| Fertility After Cancer | Stupid Cancer | | | | | | Webinar | | |
| ‘Having Children After Breast Cancer’ | Susan G. Komen Foundation | | | | | | Website | | |
| Fertility and Family Planning | Young Survival Coalition | | | | | | Website | | |
| **Women-targeted Websites** |  | | |  | | | |  | |
| **Company** | **Focus** | | | | **Website** | | | | |
| bloomlife | Maternal health | | | | www.bloomlife.com | | | | |
| Cyrcadia Health | Breast wellness monitoring | | | | www.cyrcadiahealth.com/ | | | | |
| Fertility Focus | Female fertility | | | | https://fertility-focus.com/ | | | | |
| OvuSense | Ovulation tracking | | | | www.ovusense.com/us/ | | | | |
| FertilityIQ | Fertility and family-building education | | | | www.fertilityiq.com | | | | |
| Flo Health | Menstrual cycle education | | | | https://flo.health/ | | | | |
| inne | Female fertility; hormone testing | | | | https://inne.io/ | | | | |
| Kindbody | Fertility care | | | | www.kindbody.com | | | | |
| kindara | Fertility tracking | | | | www.kindara.com | | | | |
| Maven | Women’s health care | | | | www.mavenclinic.com | | | | |
| Modern Fertility | Female fertility; hormone testing | | | | www.modernfertility.com | | | | |
| Mojo | Female fertility care | | | | www.mojofertility.co | | | | |
| robyn | Fertility, pregnancy, and postpartum support | | | | https://wearerobyn.co/ | | | | |
| univfy | Female fertility; IVF | | | | www.univfy.com | | | | |

PtDA=Patient Decision Aid

IVF=In vitro fertilization

^a^Lists informed by reviews by Speller et al.,[10] Speller et al.,[11] and Mahmoodi et al.[12]

^b^All patient decision aids reviewed were for women newly diagnosed with cancer to support decisions about fertility preservation prior to treatment; family-building topics were often also covered.
