# Supplementary material for: A Decision Aid Intervention for Family Building After Cancer: Developmental Study on the Initial Steps to Consider When Designing a Web-Based Prototype
Source: JMIR Form Res. 2021 Jan 22;5(1):e20841. doi: 10.2196/20841 (PMC7864768; doi:10.2196/20841)
Supplement: Multimedia Appendix 2 [file formative_v5i1e20841_app2.docx]

**Appendix 2.** Discovery worksheet for initial website design.^a^

| **Question** |
| --- |
| 1. Describe in one sentence what you want this resource to be. |
| 1. Describe in one sentence why you are building this website. |
| 1. Identify 3 websites that you would like to emulate in design, usability, or “look and feel.” Provide one to two sentences about each to explain why you have picked it. |
| 1. Stylistically, how would you want someone to describe your website? |
| 1. If you landed on the website as a first-time user, what would be most important to see? |
| 1. What 3-5 words would you like to overhear someone using to describe the website? |
| 1. What are your 3-5 key target audience demographics in order of importance? |
| 1. What sections or pages are most important to highlight on the website? |
| 1. What are 3-5 goals the website should accomplish or improve on in terms of user experience or design? |
| 1. What happens to this website in one year? In two years? What is your idea about how this website will evolve, if at all? |

^a^ The worksheet was developed by Radish Lab, a creative design agency.
